# The Allergic Phenotype of Children and Adolescents with Selective IgA Deficiency: A Longitudinal Monocentric Study

**DOI:** 10.3390/jcm11195705

**Published:** 2022-09-27

**Authors:** Bianca Laura Cinicola, Giulia Brindisi, Martina Capponi, Alessandra Gori, Lorenzo Loffredo, Giovanna De Castro, Caterina Anania, Alberto Spalice, Cristiana Alessia Guido, Cinzia Milito, Marzia Duse, Isabella Quinti, Federica Pulvirenti, Anna Maria Zicari

**Affiliations:** 1 Department of Maternal Infantile and Urological Sciences, Sapienza University of Rome, 00161 Rome, Italy; 2 Department of Molecular Medicine, Sapienza University of Rome, 00161 Rome, Italy; 3 Department of Translational and Precision Medicine, Sapienza University of Rome, 00161 Rome, Italy; 4 Department of Clinical, Internal Medicine, Anesthesiology and Cardiovascular Sciences, Sapienza University of Rome, 00161 Rome, Italy; 5 Department of Developmental and Social Psychology, Sapienza University of Rome, 00161 Rome, Italy; 6 Department of Internal Medicine and Infectious Diseases, Regional Reference Centre for Primary Immune Deficiencies, Azienda Ospedaliera Universitaria Policlinico Umberto I, 00161 Rome, Italy

**Keywords:** SIgAD, inborn errors of immunity, primary immunodeficiency, allergy, asthma, infections, autoimmunity

## Abstract

Background: Selective IgA deficiency (SIgAD) is the most common inborn error of immunity. The exact prevalence and pathogenesis of allergy in SIgAD have not yet been defined. We aimed to describe the prevalence and the characteristics of allergy in pediatric SIgAD subjects, evaluate the association between allergy and other comorbidities, and define the immune phenotype of allergic and non-allergic patients. Methods: Clinical and immunological data from 67 SIgAD patients were collected over a 13-year period at a single center. Patients’ characteristics were analyzed according to the presence of allergy. Results: Allergy was diagnosed in 34% of SIgAD patients, with a median age at allergy diagnosis of 8 years. Allergy was the second-most-common clinical manifestation, following recurrent respiratory infections. Among the allergic group, 74% had rhinitis, 30% asthma, 30% atopic dermatitis, and 22% food allergy; one out of three had more than one allergic manifestation. SIgAD patients showed more frequent transitory lymphopenia and a lower count of CD19+ at diagnosis than at last FU. However, compared to non-allergic subjects, allergic patients did not differ in their immune phenotype, number and severity of infections, or increased autoimmunity. Conclusions: In our longitudinal study, compared to non-allergic SIgAD patients, those with allergies did not present a more severe immune defect or complex clinical phenotype. However, evaluation and early identification of allergy in the context of SIgAD assessment, both at diagnosis and during FU, and definition of a proper management are important to prevent complications and improve the patient’s quality of life.

## 1. Introduction

Selective IgA deficiency (SIgAD) is classified as an inborn error of immunity (IEI) with antibody defects, with an estimated incidence ranging from 1:3000 to 1:150 [1,2]. Diagnosis is made when IgA serum levels are equal to or below 7 mg/dL with normal IgG and IgM levels in patients older than four years of age with an otherwise normal immune system [3,4]. When serum IgA levels are more than 7 mg/dL but <2 standard deviations (SD) for age, it is defined as partial or probable IgA deficiency [5]. The deficiency is sporadic, but autosomal dominant or recessive inheritance is observed in a variable percentage of cases (7.5–20%) [6,7]. In addition, in several cases, SIgAD can evolve into common variable immunodeficiency (CVID), a IEI with severe hypogammaglobulinemia and recurrent infections, evolving in lung damage and risk of malignancies [8].

Symptomatic patients account for 10–35% of cases, with clinical phenotypes ranging from a mild to a more severe course, while other patients can be asymptomatic and the diagnosis incidental [2,3]. 

Secretory IgA has a role in maintaining mucosal homeostasis and regulating mucosal immunity on respiratory and gut surfaces [9]. In SIgAD, although the underlying pathogenetic mechanism is not yet fully elucidated, the reduction of serum and secretory IgA may lead to colonization and penetration by pathogenic bacteria and also promote the passage of aeroallergens and food antigens, making SIgAD patients more prone to recurrent infections, autoimmunity, and allergies [10,11]. 

Recurrent infections, especially in the respiratory and gastrointestinal tracts, have been reported as the most common clinical finding leading to the initial SIgAD diagnosis [12]. However, SIgAD is also frequently associated with allergy and various atopic manifestations, including allergic rhino-conjunctivitis, asthma, food allergy, and atopic dermatitis (AD) [13]. In some cases, allergy is the first symptom or the only manifestation of the disease [14] and up to 25% of patients can be diagnosed during an allergology work-up [11]. Some authors hypothesized that allergic diseases are more common in patients diagnosed with SIgAD than in the general population [15,16,17]. However, there is still controversy about the true prevalence of allergy in SIgAD patients, which may vary by study population from 13% to 84% [18].

The mechanism by which mucosal IgA defect might promote allergic inflammation is unclear. Increased mucosal permeability with augmented levels of circulating allergens has been hypothesized [10,11]. Moreover, the decreased levels of monomeric serum IgA might contribute, resulting in a defective ability to induce ITAMi signaling, causing overactivation of the immune system and then inflammation [19]. Furthermore, atopy and IgA defect might also be the simultaneous effect of the deficiency of the TGF-beta response since TGF-beta can induce IgA synthesis and inhibit proliferation of Th 2-cells, which are involved in the pathogenesis of allergic reactions [20,21]. Lastly, the lack of the protective function of allergen-specific IgA might also have a role in facilitating allergy development [22,23,24].

The role of allergy to airborne allergens in the exacerbation of respiratory infective manifestations in SIgAD patients also remains to be defined. The chronic inflammation of the airways may facilitate the adherence of pathogens to the respiratory epithelium, as occurs in asthma [25,26]. At the same time, respiratory infections might, in turn, trigger the recurrence of asthma [27]. Moreover, it is unclear whether SIgAD subjects with recurrent infections have an increased risk for allergic disorders compared with paucisymptomatic patients and vice versa [28,29].

The prognosis of SIgAD patients is generally good, but the simultaneous presence of infections, allergies, and autoimmune diseases can result in heterogeneous outcomes and quality of life [6]. Due to the high variability of the clinical picture, it is necessary to identify early SIgAD patients with more complex clinical phenotypes or those at high risk for progression to more severe antibody defects to prevent the development of chronic comorbidities and maintain quality of life.

In this monocentric longitudinal study, we aimed to describe the clinical course of a cohort of pediatric patients with SIgAD, focusing on the allergic phenotype. In particular, we aimed to provide the prevalence of allergic manifestations in a cohort of children and adolescents with SIgAD and define the type of sensitization, treatment prescribed, and evolution of the allergic manifestations over time, giving insight into the management of allergy. Moreover, we aimed to investigate possible differences in the immune phenotype of patients with and without allergy and correlations between allergy and infectious manifestations of SIgAD.

## 2. Materials and Methods

*Study design.* A longitudinal study on patients aged < 18 years diagnosed with partial or absolute SIgAD was conducted over 13 years, from 2008 to 2021. Patients evaluated for selective IgA deficiency at the Pediatric Immunology Unit of Policlinico Umberto I (Sapienza University of Rome, Rome, Italy) were placed in a standardized protocol, including an annual clinical examination and laboratory workup. At the end of the study period, patients aged < 4 years and those who did not fulfill the criteria for absolute or partial SIgAD at the last FU (due to improvement of IgA values or decrease in IgG and IgM levels) were excluded from the analysis. Absolute SIgAD diagnosis was made according to the ESID criteria [4] (serum IgA level < 7 mg/dL; age > 4 years; normal IgG and IgM serum levels confirmed by a further blood immunoglobulin test). Children with decreased IgA levels less than 2 SD below normal age-adjusted values but >7 mg/dL were classified as partial SIgAD [5]. Patients turning 18 years terminated the study since they were transferred to an adult care center for IEIs.

Data collected at the enrollment visit included: personal data, family pedigree, and relevant clinical features including previous infections, autoimmune diseases, and allergic diagnosis; lab data collected included whole blood count (WBC), immunoglobulin (Ig) levels (IgG, IgA, IgM, and IgG subclasses), and flow cytometric evaluation of peripheral T- and B-lymphocyte percentages (anti-CD3, anti-CD4, anti-CD8, anti-CD19, and anti-CD16/56). The annual workup included chart reviews collecting annual numbers and types of infections, antibiotic cycles, new diagnoses of autoimmune or allergic diseases, allergic symptoms, and concomitant treatment. WBC and Ig levels (IgG, IgA, IgM) were collected yearly. Flow cytometric evaluation of peripheral T- and B-lymphocyte percentages and IgG subclasses were performed biannually, with the most recent being included in the study analysis. The aim of the study was to analyze the prevalence of allergic manifestations in a pediatric cohort of patients with SIgAD, defining the type of sensitization, treatment prescribed, and evolution of allergic manifestation over time. Moreover, we analyzed if associated allergies might affect the SIgAD clinical and immunological phenotype and vice versa.

The study protocol was approved by the Ethical Review Committee of Sapienza, University of Rome, Italy (Prot. 0259/2022, 1 April 2022). The study was performed in accordance with the Good Clinical Practice guidelines and the most recent version of the Declaration of Helsinki.

*Allergy diagnosis.* Patients were grouped based on allergy diagnosis. We included in the group *SIgAD with allergy* those participants with at least one atopic manifestation among (a) atopic dermatitis (AD), (b) food allergy, (c) allergic rhinitis, and (d) allergic asthma. We also included in the group of allergic SIgAD patients whose diagnosis of allergy was previous to SIgAD diagnosis. For the definition of allergic rhinitis, we considered the correspondence between the sensitization found and the nasal symptoms reported. For patients with isolated AD, at least one positivity to skin prick test (SPT) or the presence of specific serum IgE levels was required. Allergic asthma, allergic rhinitis, and food allergy were defined according to GINA [30], ARIA [31], and EAACI guidelines [32]; AD was defined according to Hanifin and Rajka criteria [33]. SPTs (Lofarma, Milan, Italy) were performed using extracts of the most common pollen present in Italy (parietaria, grass pollen, olive tree, cypress, pine, plane tree, and birch). In addition, we used house-dust mite (*Dermatophagoides pteronyssinus* (DPT) and *Dermatophagoides Farinae* (DF)), mold (alternaria), dog and cat epithelium extract, and food extracts (lactoglobulin, alpha-lactalbumin, casein, albumen, egg yolk, wheat, fish, and soy). The panel of food allergens chosen was based on the highest incidence of food allergies in our country and varied according to the clinical symptomatology reported by the patient after contact with a specific allergen. The tests were performed according to EAACI guidelines. Histamine hydrochloride (10 mg/mL) was used as a positive control and glycerol solution as a negative one. The skin was pricked using Morrow Brown needles. The wheal was evaluated after 15 min, and a wheal of ≥3 mm was considered positive [34].

*Statistical analysis.* The primary analysis of the observational study was to investigate clinical and laboratory characteristics in two groups defined as *SIgAD with allergy* and *SIgAD without allergy.* Continuous variables were described using median and interquartile ranges, and categorical variables using frequencies and percentages. Immunological and clinical variables were compared between SIgAD patients with allergy and SIgAD patients without allergy. Values were compared by the non-parametric two-tailed Mann–Whitney or Fisher’s test. The significance threshold was set at *p* less than 0.05. Statistical analysis was performed with SPSS 18.0 software for Windows (SPSS, Chicago, IL, USA).

## 3. Results

*Patient cohort.* We included in the analysis 67 children and adolescents with SIgAD (31 females, 45%), the median age at the last FU was 9 years (IQR 7–13.5), followed during a 13-year period (from 2008 to 2021) for a total of 262 patient-years. The median FU per participant was 3 years (IQR 1–6.5). The median age at diagnosis was 5.5 (IQR 3–8). A positive family history of primary antibody defect/hypogammaglobulinemia was identified in nine participants (14%), of which seven patients belonged to three clusters of brothers. Baseline patients’ characteristics are summarized in Table 1. Reasons for performing serum immunoglobulin assay included recurrent infections (71%), screening for celiac disease (11%), allergy work-up (5%), family history of SIgAD or hypogammaglobulinemia (5%), and poor growth (3%) (Table 1). One patient with a progressive reduction of the serum IgG and IgM levels during the FU consistent with the diagnosis of CVID was excluded from the analysis. Two patients who reached IgA-normal serum levels for their age during the FU were also excluded.

*Serum immunoglobulin levels and lymphocyte subsets.* At diagnosis, IgA median value was 4 (IQR 2–5) mg/dL. Serum IgG, IgM, and IgG subclass levels were normal for all patients. Fifty-nine out of 67 (88%) fulfilled the criteria for absolute SIgAD, whereas the remaining eight patients (12%) had partial SIgAD (serum IgA 10 mg/dL (IQR 8.5–10.5) (Table 1). During the FU, six patients with partial SIgAD progressively reduced the IgA serum levels to <7 mg/dL, whereas three patients with absolute SIgAD increased the IgA serum levels over 7 mg/dL. Then, at the last FU, 62 patients (93%) were classified as having absolute SIgAD and five patients (7%) as having partial SIgAD. At the end of the study period, no patient showed associated IgG subclasses defect.

Peripheral lymphocyte subset evaluation was performed at diagnosis and biannually during the FU. Based on the age-related reference range, lymphopenia and low count of CD19+ were more frequently seen at SIgAD diagnosis than at last FU (65% vs. 1.5%, *p* < 0.0001 and 57% vs. 11%, *p* < 0.0001, respectively). However, no patients displayed a severe reduction of lymphocytes count or severe abnormalities of B and T cells phenotype, with only two children showing the CD4+ count below 400 cell/mm^3^ at diagnosis and no patients having the CD19+ count below 100 cell/mm^3^ during the study time (Table 1).

**Figure 1:**
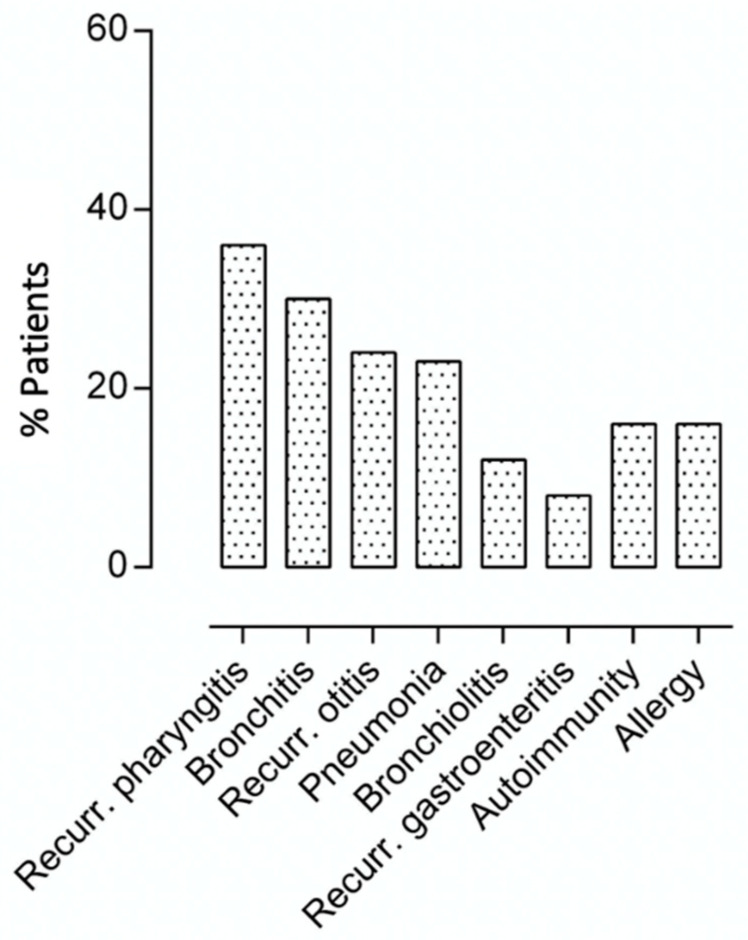
Prevalence of clinical manifestations at SIgAD diagnosis.

*Clinical manifestations at diagnosis and during the follow-up.* The prevalence of clinical manifestations at diagnosis is shown in Figure 1. Respiratory infections were the most frequent clinical features, with the upper respiratory tract being recorded in 43% (*n* = 29) of patients. In detail, recurrent otitis and pharyngotonsillitis (>3 episodes per year) were recorded in 24% and 36% of patients, respectively. Lower respiratory tract infections were also observed, with recurrent bronchitis (>2 episodes/year) and pneumonia (at least one episode before SIgAD diagnosis) recorded in 30% (*n* = 20) and 23% (*n* = 15) of patients. Twelve percent (*n* = 8) had a past medical history of bronchiolitis and 8% (*n* = 5) reported having recurrent gastroenteritis (>2 episodes/year). Sinusitis and recurrent urinary infections were not reported by the patients. Of note, 24% of subjects (*n* = 16) had a positive medical history for hospital admission for infection before the SIgAD diagnosis.

As shown in Table 2, during FU, pharyngitis was the most frequently reported infection, being recorded in 20.3% of patients, followed by bronchitis (20.3%), gastroenteritis (12%), otitis media (17.2%), and urinary infections (6.3%). Only two patients experienced pneumonia during the follow-up (3.1%). Twelve percent (*n* = 8) of participants underwent adenoidectomy and/or tonsillectomy before enrollment and during the FU. Overall, 151 antibiotic courses in 33 patients were prescribed during the study time. Moreover, 25% (*n* = 17) had a diagnosis of asthma, of which 10 patients did not show any allergic sensitization. All except one were classified as intermittent asthma, the other as mild persistent.

Autoimmune manifestations were initially found in 9/67 patients (13%) (Figure 1). An additional eight SIgAD patients were diagnosed with autoimmune disorders during the study period—a cumulative autoimmune manifestation prevalence of 25%. Celiac disease was present in eight patients, thyroiditis in six patients, diabetes mellitus type 1 in one patient, and arthritis in one patient, while one patient had both thyroiditis and celiac disease. The median age at diagnosis of autoimmunity was 8.0 (IQR 3.5–9) years.

*Allergy in SIgAD.* Positive family history of allergy was identified in 57% (*n* = 38) of SIgAD participants. The prevalence of allergy in the enrolled cohort accounted for 34% (*n* = 23); 11 patients (16%) received the diagnosis of allergy before SIgAD diagnosis, and an additional 12 patients (18%) were diagnosed with the allergic disease during the study period. The median age at allergy diagnosis was 8 (IQR 3–10) years. In detail, seven patients (10%) were diagnosed with intermittent allergic asthma; 17 (25%) had allergic rhinitis, classified as mild intermittent in a single case, moderate intermittent in three cases, mild persistent in four, and moderate persistent in nine cases. Ten patients (15%) had AD, with a mild degree in six cases and moderate in four; among them, only seven patients had a positive allergic sensitization. Five subjects (7%) were diagnosed as having food allergies (Table 3).

Eight out of 23 patients had more than one allergic manifestation (Table 3). We further analyzed the time-based order of allergic diseases, finding that most patients showed the same order observed in non-SIgAD allergic patients [35], from AD and food allergy in infancy to gradual development into allergic asthma and allergic rhinitis in childhood (Table S1). Ninety-one percent (*n* = 21) of SIgAD patients with allergy were sensitized to inhalants and 22% (*n* = 5) to food. Seventy-eight percent (*n* = 18) were sensitized to more than one allergen. The most common allergic sensitivities in our cohort were dust mite, followed by grass pollen and olea. Allergen sensitizations identified are reported in Table 4. In the cohort examined, SIgAD patients with asthma were more likely also to have AD (57% vs. 13%, *p* = 0.0146).

The treatment received for allergies is summarized in Table S2. It reflects the underlying allergic pathology and was based on allergy guidelines. Of note, two patients received a three-year desensitizing therapy for dust mite and grass for the treatment of rhinitis symptoms, with clinical improvement. Furthermore, one patient with chronic allergic rhinitis and chronic urticaria had been treated with omalizumab.

A comparison between SIgAD children and adolescents with and without allergy is summarized in Table 5. When compared to patients without allergies, SIgAD patients with allergies did not differ in age at the SIgAD diagnosis but had a longer FU (2.0 (IQR 4–11) vs. 6 years (IQR 3.5–8), *p* = 0.0010). As expected, when compared to patients without allergy, SIgAD patients with allergy had higher IgE serum levels (33 UI/mL (IQR 14–55) vs. 474 UI/mL (IQR 49–1119), *p* = 0.018), whereas they did not have lower IgG, IgA, IgM, or IgG subclass serum levels either at diagnosis or during FU. SIgAD patients with allergy did not have a more severe infectious phenotype, as they did not differ from non-allergic SIgAD patients in the frequency of infectious episodes, type of infection, or need for hospitalization during the FU. Finally, allergic and non-allergic SIgAD patients had a similar incidence of autoimmune diseases (Table 5).

## 4. Discussion

The loss of secretory and serum IgA is the main immunological characteristic of SIgAD, the most common IEI [5,11]. Although respiratory infections are the prevalent manifestations reported, allergy is also part of the clinical picture of SIgAD [3,12].

There is controversy about whether allergic diseases are more common in patients diagnosed with SIgAD than in the general population [2,6]. Indeed, the prevalence of allergies in SIgAD varies by the study population [18], and studies focusing on the characterization of the allergic phenotype in these patients are scarce.

In this longitudinal monocentric study, we evaluated 67 children and adolescents diagnosed with SIgAD, aiming to describe the clinical and immunological phenotype of the allergic patients, and characterize the natural history of allergic manifestations in this population.

Although we confirmed the recurrence of infections as the main reason for the initial immunological assessment [36,37], in our cohort, a co-existing allergic manifestation was recorded in 16% at the time of SIgAD diagnosis, making allergy the second most frequent clinical feature at initial evaluation. Of note, allergies were diagnosed both at SIgAD diagnosis and during follow-up, leading to a cumulative prevalence of allergy of 34%.

Data on the prevalence of allergy in SIgAD reported by studies from different countries are highly heterogeneous, ranging from 13 to 84% [14,38], suggesting an effect of ethnic background. In support of this hypothesis, the prevalence of allergy recorded in our study was in line with what had been reported in previous Italian pediatric SIgAD cohorts [36,37,39] and higher than what had been reported for Italian healthy pediatric subjects [40]. In detail, the prevalence of asthma, allergic rhinoconjunctivitis, and atopic dermatitis in our cohort was 10%, 25%, and 15%, respectively, while in the Italian pediatric population it is low (asthma: 7.9–8.4%, rhinoconjunctivitis 6.5–15.5%, atopic dermatitis 7.7–10.1%) [40]. These observations also emphasize the need for screening patients for allergies after SIgAD has been established.

Compared to other SIgAD cohorts, differently from previous studies in which asthma was the most common manifestation or had a prevalence similar to rhinitis [14,15,28,29,36,41,42,43], in our cohort allergic rhinitis was the main finding, with persistent symptoms in most of them. However, independently from allergic etiology, asthma is a frequently encountered clinical feature in SIgAD, both at diagnosis and during FU [44]. In our sample, one out of four patients was asthmatic, with mild symptoms in all cases except one, well controlled by inhalant therapy. Comparison with data on atopic dermatitis and food allergy were more difficult, due to high variability of frequencies reported. In our cohort, we reported 10 patients with AD, seven with allergic sensitization. As in the general population, the prevalence of AD varies in SIgAD patients, possibly due to heterogeneity in diagnostic criteria for AD among studies and ethnic backgrounds [45]. Two specific studies of SIgAD patients recorded a prevalence of AD in 4.6% [46] and in 57.84% of cases [47], although in the second study only 10% of those patients had elevated IgE. Concerning food allergy, data are conflicting—two studies reported an incidence of 29% [16] and 22% [8], while others recorded a lower incidence than ours [28,29,41]. This variability could be related to geographic differences and cultural and dietetic habits and also to a difference in diagnostic methods among studies [48].

More than one out of three allergic SIgAD patients had more than one allergic manifestation. In patients with SIgAD the recorded order of appearance, the so-called atopic march, generally proceeded from DA/food allergy to asthma/rhinitis, as in immunocompetent children [49]. We found a significant correlation between AD and asthma. It has been hypothesized that diverse pathways initiated by AD might modulate specific biomarkers associated with the development of asthma [50] and that skin-barrier impairment as well as decreased airway epithelial integrity may play a role in sensitization and immune modulation [51].

Concerning the type of sensitization, the main allergens identified were dust mites, followed by grass pollen, olive tree, animal epithelium, and cypress. Dust mite positivity was present in most SIgAD patients with allergic asthma. Our observation confirmed dust mite as the most common allergen identified for allergic patients with SIgAD, apart from the differences in seasonal allergens observed in different countries [14,37,52].

Since published data on infection severity in children with SIgAD were contrasting [28,29], one of the study’s goals was to assess if respiratory allergic symptoms might represent a risk factor for SIgAD-related comorbidities, including respiratory tract infections. In our cohort, recorded prevalence and type of infections were similar in allergic and non-allergic SIgAD patients. Of note, SIgAD patients with allergies had a longer follow-up when compared to those without allergies, suggesting closer surveillance and prompt treatment to prevent complicated infections, possibly minimizing the differences between groups.

In immunocompetent individuals, asthma is known to be a worsening factor in the number of respiratory infections [26,27,53]. Moreover, asthma was found to be less responsive to standard treatments in patients with humoral defects and tended to become chronic [54]. However, data on the relationship between asthma and infection in SIgAD are not conclusive [28,29]. We observed a similar rate of infections in SIgAD patients with and without asthma, possibly due to the mild respiratory-disease severity of lung involvement and good control of symptoms by treatment. Indeed, it is known that if well-controlled, asthma and respiratory allergic symptoms are not a risk factor for the recurrence and severity of infections [55,56]. However, asthmatic patients with SIgAD were more likely to have concurrent AD, suggesting a more complex allergic phenotype and supporting the role of inflammation initiated by AD in the development of asthma [51].

Previous studies on SIgAD patients revealed normal WBC, decreased levels of peripheral class-switched memory B cells which cannot differentiate into IgA-secreting plasma cells, and reduced frequencies of T-reg cells in patients with autoimmunity [57,58,59,60]. In this cohort, no patients displayed a severe reduction of neutrophils or lymphocytes, or severe abnormalities of B- and T-cell phenotypes. However, compared to the last follow-up, SIgAD patients showed more frequent transitory lymphopenia and a lower count of CD19+ at the diagnosis time. A comprehensive peripheral B- and T-cell phenotyping analysis is needed to better interpret this observation. Allergic SIgAD patients did not have a more severe immune or IgA defect, as immunoglobulin serum levels were similar in allergic and non-allergic patients, except for IgE serum levels that were higher in those with allergies. The ability to produce IgE demonstrates the integrity of a Th2 response in SIgAD patients and supports the hypothesis of a selective defect in class-switch recombination (CSR) and differentiation into only IgA-secreting plasma cells [10].

As for other IEIs, treatment of allergic diseases in SIgAD patients may be challenging, due to the increased risk of infection and the coexistence of autoimmunity in some individuals. Recommendations by the American Academy of Allergy, Asthma & Immunology (AAAAI) and the American College of Allergy, Asthma & Immunology (ACAAI) firmly stated the need to treat allergy in those living with IEIs, including SIgAD, since allergic inflammation might facilitate the development of respiratory tract infections [5]. Following these guidelines, treatment prescribed in our cohort did not differ from the standard modalities based on the underlying allergic disease and included avoidance of allergens, medication, immunotherapy, and anti-TH 2 biologics such as omalizumab. An increased risk of infections or the need for changes in standard care for SIgAD patients was not observed to treat allergic manifestations.

The main limitation of this study was the lack of a control group. An age-matched control group was not included due to difficulties in enrolling healthy children in a longitudinal study, however we compared our results with those of population-based studies.

## 5. Conclusions

In our longitudinal study, SIgAD children with an allergy do not appear to have a more complex clinical or immune phenotype than non-allergic children. In particular, allergy in our cohort did not seem to be associated with an increased risk of other comorbidities, especially respiratory infection. In any case, most of our patients have mild allergies and good control of allergic symptoms, which could result in a reduced risk of infection recurrence and severity. Thus, evaluation and early identification of allergy in the context of SIgAD assessment, both at diagnosis and during follow-up, enables the establishment of adequate prevention and targeted therapies to limit the onset of chronic disease and associated complications and improve the quality of life of SIgAD patients and their families.

## Figures and Tables

**Table 1:** Characteristics of 67 enrolled children and adolescents with SIgAD.

	SIgAD Patients (*n* = 67)	
Age at last FU, years, median (IQR)	9 (7–13.5)	
Female, *n* (%)	31 (45)	
Absolute SIgAD, *n* (%)	62 (93)	
Time of FU, year, median (IQR)	3 (1–6.5)	
Family history of hypogammaglobulinemia, *n* (%)	9 (14)	
Reason for first serum immunoglobulin assessment, *n* (%)		
Recurrent infections	47 (71)	
Screening for celiac disease	7 (11)	
Allergy work-up	3 (5)	
Family history of SIgAD or hypogammaglobulinemia	3 (5)	
Growth delay	2 (3)	
Other	3 (5)	
	**At Diagnosis**	**At Last FU**
WBC		
Neutrophils ×10^9^/L, median (IQR)	2.9 (2.4–3.7)	3.4 (2.7–3.9)
Lymphocytes ×10^9^/L, median (IQR) **	2.1 (1.9–2.8)	2.3 (2.0–2.8)
Low number of lymphocytes *, *n* (%)	21 (65)	1 (1.5)
Immunoglobulins serum levels		
IgA mg/dL, median (IQR)	4 (2–5)	3.4 (2–5)
IgG mg/dL, median (IQR)	1335 (1140–1650)	1275 (1156–1567)
IgM mg/d, median (IQR)	102 (70–124)	100 (72–111)
IgE U/mL, median (IQR)	49 (19–480)	54 (22–459)
B and T cell peripheral phenotype		
Low CD3+ count *, *n* (%)	13 (23)	3 (7.5)
CD3+/mm^3^, median (IQR)	1699 (1406–2074)	1708 (1415–2380)
Low CD4+ count *, *n* (%)	12 (22)	3 (9)
CD4+/mm^3^, median (IQR)	840 (695–1121)	909(691–1206)
Low CD8+ count *, *n* (%)	4 (7)	3 (8)
CD8+/mm^3^, median (IQR)	515 (530–793)	672 (524–994)
Low CD19+ count *, *n* (%)	25 (57)	4 (11)
CD19+/mm^3^, median (IQR) **	329 (262–537)	321 (263–615)

* According to age-related reference range. Abbreviations: FU follow-up; SIgAD, selective IgA deficiency; WBC white blood cells. ** Diagnosis vs. last follow up: *p* < 0.0001.

**Table 2:** Incidence of infectious manifestations in 67 patients with SIgAD during follow-up.

	Patients		Episodes during FU	Episodes/Year	
	*n*	%	*n*	Median	IQR
**Pharyngitis**	13	20.3%	90	0.25	(0–1)
**Bronchitis**	13	20.3%	31	0.00	(0–0)
**Gastroenteritis**	12	18.8%	29	0.00	(0–0)
**Otitis**	11	17.2%	26	0.00	(0–0)
**Urinary infections**	4	6.3%	22	0.00	(0–0)
**Pneumonia**	2	3.1%	2	0.00	(0–0)
**Antibiotic courses**	33	51.6%	151	0.00	(0–1.25)

Abbreviation: FU, follow up.

**Table 3:** Allergic manifestations in the 23 SIgAD children and adolescents with allergy.

	SIgAD with Allergy (*n* = 23)
Age at allergy diagnosis, median (IQR)	8 (3–10)
Family history of allergy, *n* (%)	38 (57)
Atopic manifestations, *n* (%)	
Allergic asthma	7 (30)
Allergic rhinitis	17 (74)
Atopic dermatitis	7 (30)
Food allergy	5 (22)
More than one allergic manifestation, *n* (%)	8 (35)
Sensitization for more than one allergen, *n* (%)	18 (78)

**Table 4:** Allergen sensitization in the 23 SIgAD children and adolescents with allergy.

	SIgAD with Allergy (*n* = 23)
SPT for inhalants, *n* (%)	21 (91)
DPT	14 (60)
DF	12 (52)
Alternaria	3 (13)
Parietaria	1 (4)
Grass pollen	8 (35)
Olive tree	6 (26)
Cypress	1 (4)
Pine	0
Plane tree	0
Birch	0
Dog epithelium	2 (9)
Cat epithelium	1 (4)
SPT for food, *n* (%)	5 (22)
Milk, lactoglobulin	3 (13)
Milk, alfa-lattoalbumin	4 (17)
Egg, yolk	3 (13)
Egg, white	3 (13)
Soy	0
Wheat	0
Fish	0
Milk, casein	0

Abbreviation: SPT, skin prick test.

**Table 5:** Clinical and immunological features of SIgAD children and adolescents grouped according to the allergy diagnosis at the last follow-up.

	SIgAD without Allergy *n* = 44	SIgAD with Allergy *n* = 23	*p* Value
Age at SIgAD diagnosis, years, median (IQR)	5 (3–7.2)	8 (4.5–9)	0.397
Years of FU, median (IQR)	2 (11–4)	6 (3.5–8)	0.001
Ig levels at SIgAD diagnosis, median (IQR)			
IgA mg/dL	4 (2–5)	4 (2–5.5)	0.242
Igg mg/dL	1330 (1115–1520)	1500 (1165–1705)	0.231
IgM mg/dL	109 (73–124)	77 (47–118)	0.404
IgE mg/dL	33 (14–55)	474 (49–1119)	0.018
Clinical manifestations at SIgAD diagnosis			
Hospital admission for infections, *n* (%)	10 (23)	6 (30)	0.547
Recurrent otitis, *n* (%)	12 (27)	4 (18)	0.375
Pneumonia, *n* (%)	11 (25)	4 (18)	0.756
Recurrent bronchitis, *n* (%)	14	6 (27)	0.782
Bronchiolitis, *n* (%)	6 (14)	2 (9)	0.708
Recurrent gastroenteritis, *n* (%)	3 (7)	2 (9)	1.000
Autoimmunity, *n* (%)	17 (39)	11 (48)	0.201
Low lymphocyte count * at last FU	1 (3)	0 (0)	1.000
Low CD3+ count *, *n* (%) at last FU	2 (8)	1 (7)	1.000
Low CD4+ count *, *n* (%) at last FU	2 (10)	1 (7)	0.550
Low CD8+ count *, *n* (%) at last FU	2 (9)	1 (7)	1.000
Low CD19+ count *, *n* (%) at last FU	2 (9)	2 (17)	0.594
Rate of respiratory infections during FU, median (IQR)	0.17 (0–1.5)	0 (0.075)	0.253

* According to age-related reference range. Abbreviations: FU, follow-up; SIgAD, selective IgA deficiency.

## Data Availability

The data presented in this study are available on request from the corresponding author. The data are not publicly available due to privacy issues.

## Supplementary Materials

The following supporting information can be downloaded at: https://www.mdpi.com/article/10.3390/jcm11195705/s1, Table S1: Atopic march in patients with more than one allergic manifestation; Table S2: Treatment of atopy in SIgAD.
