# Supplementary material for: The Allergic Phenotype of Children and Adolescents with Selective IgA Deficiency: A Longitudinal Monocentric Study
Source: J Clin Med. 2022 Sep 27;11(19):5705. doi: 10.3390/jcm11195705 (PMC9573591; doi:10.3390/jcm11195705)
Supplement: Supplementary file 1 [file jcm-11-05705-s001.zip › jcm-1889101-supplementary.pdf]

Journal of
Clinical Medicine

MDPI

Table S1. Atopic March in patients with more than one allergic manifestation.

Sex/Age	Allergic symptoms in order of appearance/age of onset
M/18y	Food allergy/1y<br>Asthma/3y
M/12y	Food allergy/1,6y<br>Allergic rhinitis/5y
M/12y	DA/0,6y<br>Food allergy/3y<br>Allergic rhinitis/8y
M/9y	DA/0,3y<br>Allergic asthma/5y<br>Allergic rhinitis/5y
M/8y	Asthma/6y<br>Allergic rhinitis/8y
M/7y	DA/0,3y<br>Food allergy/0,4y<br>Allergic Asthma/5y
M/16y	DA/0,3y<br>Allergic Asthma/5y<br>Allergic Rhinitis/11y
M/15y	DA/1y<br>Allergic Rhinitis/10y
M/17y	Asthma and Allergic Rhinitis/8y

Abbreviation: DA atopic dermatitis.

Table S2. Treatment of atopy in SIgAD.

	**SIgAD n=67**
**Allergic Rhinitis, n (%)**	17 (25)
Steroid nasal sprays	14 (20)
Antihistamines	10 (15)
Desensitization	2 (3)
**Allergic Asthma, n (%)**	7 (10)
SABA on-demand	7 (10)
Corticosteroid inhalers	5 (7)
Antileukotrienes	4 (6)
**Atopic Dermatitis, n (%)**	10 (15)
Topical steroids	4 (6)
Emollient and moisturizing creams	10 (15)

Abbreviation: SABA, Short-Acting Beta Agonists, SIgAD Selective IgA deficiency.
